# Unemployment and health selection in diverging economic conditions: Compositional changes? Evidence from 28 European countries

**DOI:** 10.1186/s12939-015-0258-8

**Published:** 2015-11-04

**Authors:** Kristian Heggebø, Espen Dahl

**Affiliations:** Oslo and Akershus University College, Faculty of Social Sciences, PB 4 St. Olavs Plass, N-0130, Oslo, Norway

**Keywords:** Unemployment, Health selection, Economic crisis, Europe

## Abstract

**ᅟ:**

Unemployment and health selection in diverging economic conditions: Compositional changes? Evidence from 28 european countries.

**Introduction:**

People with ill health tend to be overrepresented among the unemployment population. The relationship between health and unemployment might, however, be sensitive to the overall economic condition. Specifically, the health composition of the unemployment population could change dramatically when the economy takes a turn for the worse.

**Methods:**

Using EU-SILC cross sectional data from 2007 (pre-crisis) and 2011 (during crisis) and linear regression models, this paper investigates the relationship between health and unemployment probabilities under differing economic conditions in 28 European countries. The countries are classified according to (i) the level of and (ii) increase in unemployment rate (i.e. >10 percent and doubling of unemployment rate = crisis country).

**Results:**

Firstly, the unemployment likelihood for people with ill health is remarkably stable over time in Europe: the coefficients are very similar in pre-crisis and crisis years. Secondly, people with ill health have experienced unemployment to a lesser extent than those with good health status in the crisis year (when we pool the data and compare 2007 and 2011), but only in the countries with a high and rising unemployment rate.

**Conclusion:**

The health composition of the unemployment population changes significantly for the better, but only in those European countries that have been severely hit by the current economic crisis.

## Introduction

Europe is currently struggling with a deep and long-lasting economic downturn, commonly referred to as “the Great Recession”. The probably most important consequence of the recession has been the large increase in unemployment rates. In the 28 EU member countries as a whole, the unemployment rate increased from 6.8 percent in the start of 2008 to 11.0 percent in 2013 [1]. When the economy takes a turn for the worse – and overall unemployment rates increase rapidly – *the composition of the unemployment population* will supposedly change. In this situation, employers will have to fire healthy and productive employees that would otherwise have kept their jobs, and this will probably lead to a kind of “positive selection” into unemployment. For instance, people with higher educational levels could end up losing their jobs to a similar extent as those holding lower education. Likewise, people with good health status could experience unemployment to a similar (or even higher) degree, compared to people with ill health. The relationship between health status and unemployment in changing economic conditions is the topic of the current study, and we ask the following research question: *Do people with ill health experience unemployment to a lesser extent than those with good health during the economic downturn in Europe?*

That there is a statistical relationship between ill health and heightened unemployment likelihood is a well-established empirical fact, and this is due to both selective processes [2–4] and that health status deteriorates while being unemployed [5–7]. Furthermore, there is some evidence that the association between ill health and employment status could be sensitive to the overall economic condition of a country: it seems as though people with ill health struggle to re-enter the labor market in post-recessionary periods [8–10]. What is currently lacking in the existing literature on health and unemployment, however, is a clearer comparative focus, as much of the previous research on this topic has analyzed data from only one country. The current paper will try to fill this gap by investigating the unemployment likelihood for people with ill health in 28 European countries during diverging economic circumstances.

The cross-sectional part of the European Union Statistics on Income and Living Conditions (EU-SILC) data material is utilized, and linear regression models (OLS) are run. 2007 and 2011 are set as pre-crisis and crisis years respectively, and we investigate whether the relationship between ill health and unemployment probability is modified by a sudden change in the economic conditions. We add an explicit *cross-national* perspective to the research design by classifying countries according to the severity of the economic crisis: Countries in which the unemployment rate is above 10 percent in 2011, and where there was a doubling of the unemployment rate from 2007 to 2011 are classified as ‘crisis countries’. The remaining countries are classified according to the percentage change in the unemployment rate, and we differentiate between ‘mild crisis’ (2.6-5 % increase), ‘small increase’ (1–2.5 % increase) and ‘no crisis’ (<1 % increase).

### Previous research and crisis classification

#### Health and employment status

The current study will investigate whether people with ill health are more likely to be unemployed (commonly referred to as *health selection*), and to what degree the economic condition can alter this relationship. Previous studies have shown that people with ill health have a higher unemployment probability than people with good health [2–4]. Furthermore, there seems to exist a robust statistical association between health problems and a lower likelihood of having or gaining employment [11–13].

People who become unemployed could even deteriorate in health due to the stress pertaining to this adverse experience [14, 15]. Yet the empirical evidence is rather mixed on the negative causal effect of unemployment on health, where some find evidence of such a relationship [5–7], and others do not [16–19]. The relationship between health and employment status is probably of a reciprocal kind, where both health selection and health effects of unemployment is at work simultaneously [20–22].

In summary, a large body of research suggests a strong (reciprocal) relationship between ill health and employment status. Due to both selective processes and health effects of unemployment, the unemployed tends to be in worse health than the employed. Since the unemployment “penalty” for people with ill health is well established empirically, closer attention should be devoted to how the relationship *varies over time and space.* It might be the case, for instance, that certain circumstances are able to modify the negative association between health and unemployment. Consistent with this argument, a recent study finds cross-national differences in unemployment probabilities for people with ill health in Scandinavia, where health selection is most apparent in Denmark [23]. The current paper will investigate the time dimension, with an emphasis on the role of *changing economic conditions*.

#### Health selection in changing economic conditions

The relationship between health and employment status in changing economic conditions has been investigated to some extent previously, although most often using data from only one country. A British study found that people with ill health struggled to re-enter the labour market in the aftermath of economic downturns in 1973–93 [8]. A replication of this paper using a longer observational period (1973–2009) revealed similar findings [9]. Comparable patterns have been observed in Norway as well, where people reporting ill health had comparatively low employment rates after the recession in the late 1980s/ early 90s [10].

The three above-mentioned studies all investigate whether people with ill health continue to be disadvantaged *after* economic downturns. We ask a different research question: is the relationship between health and unemployment probability noticeably different *during* an economic crisis? In a similar vein, Åhs & Westerling [24] found that the differences in self-rated health between the employed and unemployed were greater when Sweden experienced high unemployment levels (in the 1990s), compared with a more “booming” economic condition. We follow the same path, but add an explicit *cross-national component* using data for 28 European countries. In addition, we investigate unemployment likelihood for people with health problems during the ongoing “Great Recession”, where the included countries differ quite extensively concerning how severe the impact of the crisis has been, as measured by national unemployment rates.

#### Country classification: severity of crisis

In the following, economic conditions are investigated along a time dimension, through the comparison of unemployment probabilities for people with ill health in a crisis and a pre-crisis year. Additionally, we use cross-national differences in the overall *severity of the crisis* to localize countries in which there was (i) no crisis at all, (ii) a small increase in the unemployment rate, (iii) a mild crisis, and (iv) a full-blown crisis.

Our reasoning is that in order for the “newly” unemployed to influence the composition of the unemployment population, two criteria must be fulfilled for the crisis to be counted as severe. First, unemployment during times of crisis must be a “mass phenomenon”, and, second, a high amount of people must recently have lost their job. Thus, we take into account both the overall unemployment *rate* and how rapidly it *increased*. Our operationalization of severe crisis goes like this: nations in which the unemployment rate was (i) over 10 percent in 2011, and (ii) where the unemployment rate doubled from 2007 to 2011 are defined as ‘crisis countries’. We admit that this classification is somewhat arbitrary, but we think it is reasonable. Countries with a continuingly high unemployment rate (but no increase) will not help us much, since we are interested in the effects of changing economic conditions. Neither are noticeable upward changes from a very low level (e.g. from 2 to 7 percent) likely to alter the unemployment population much, since being unemployed is still a rather rare event.

The years 2007 and 2011 are set as *pre-crisis* and *crisis year* respectively (more on the reasons for this choice below). Because our main interest is the potential change in the composition of the unemployment population, *overall national unemployment rate* is the most relevant crisis indicator. A crisis measure based on GDP is in this case not preferred because the unemployment rate tends to lag behind GDP changes [25]. This implies that a country could experience “jobless growth”, where the economy is improving, while the unemployment rate stays high [26], leading to a misclassification of the country.

Table 1 provides official unemployment statistics from Eurostat in 2007 and 2011. As mentioned above, countries in which the unemployment rate is (i) over 10 percent in 2011 and (ii) where the unemployment rate doubled from 2007 to 2011 are classified as ‘crisis’ (e.g. Estonia: from 4.6 to 12.3 percent). The remaining countries are classified according to the percentage change in the unemployment rate. Countries who experienced between 2.6 and 5 percent increase are classified as ‘mild crisis’ (e.g. Hungary: from 7.4 to 11.0 percent), whereas an increase between 1.0 and 2.5 percent are classified as a ‘small increase’ (e.g. the Czech Republic: from 5.3 to 6.7 percent). Countries in which there was below 1 percent increase – or even a reduction – in the unemployment rate are classified as ‘no crisis’ (e.g. Belgium: from 7.5 to 7.2 percent).

**Table 1:** Overall unemployment rate 2007 and 2011 in 28 European countries. Source: Eurostat

Country	Pre-crisis: 2007	Crisis: 2011
Crisis		
Estonia	4.6	12.3
Greece	8.4	17.9
Ireland	4.7	14.7
Latvia	6.1	16.2
Lithuania	4.3	15.4
Spain	8.2	21.4
Mild crisis		
Bulgaria	6.9	11.3
Cyprus	3.9	7.9
Denmark	3.8	7.6
Hungary	7.4	11.0
Iceland	2.3	7.1
Portugal	9.2	12.9
Slovenia	4.9	8.2
United Kingdom	5.3	8.1
Small increase		
Czech Republic	5.3	6.7
France	8.0	9.2
Italy	6.1	8.4
Slovakia	11.2	13.7
Sweden	6.1	7.8
No crisis		
Austria	4.9	4.6
Belgium	7.5	7.2
Finland	6.9	7.8
Germany	8.5	5.8
Luxembourg	4.2	4.8
Netherlands	4.2	5.0
Norway	2.5	3.3
Poland	9.6	9.7
Romania	6.4	7.2

Classification

Crisis = Doubling of overall unemployment rate and > 10 percent

Mild crisis = 2.6-5.0 percent increase

Small increase = 1.0-2.5 percent increase

No crisis = < 1.0 percent increase

*2007 EU-SILC- data not available for Croatia, Malta and Switzerland*

Estonia, Greece, Ireland, Latvia, Lithuania and Spain fulfills the two criteria stated above, and therefore represents the *crisis* countries. These six countries also stand out regarding percentage changes in the unemployment rate, varying from 7.7 in Estonia to 13.2 in Spain. There is a *mild crisis* in Bulgaria, Cyprus, Denmark, Hungary, Iceland, Portugal, Slovenia, and the U.K., and a *small increase* in the unemployment rate is evident in the Czech Republic, France, Italy, Slovakia and Sweden. Lastly, there is *no crisis* (and even decreasing unemployment) in Austria, Belgium, Finland, Germany, Luxembourg, the Netherlands, Norway, Poland and Romania. This implies that – according to our classification – there is a crisis or a mild crisis in 14 of the 28 included European countries. In the remaining half, there is only a small increase in the unemployment rate, and in three cases (Austria, Belgium and Germany) even reductions.

**Fig. 1:**
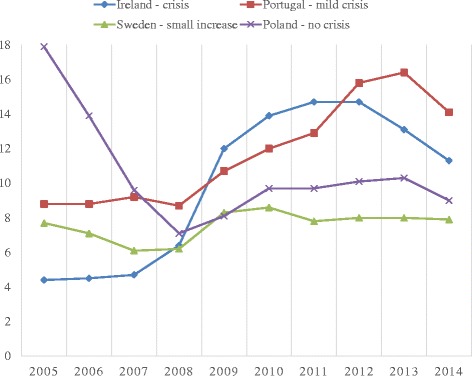
Unemployment rates 2005–2014 for Ireland (crisis), Portugal (mild crisis), Sweden (small increase) and Poland (no crisis). Source: Eurostat

Figure 1 shows the unemployment rate for Ireland, Portugal, Sweden and Poland (one country from each category), from 2005 and ten years onwards. The countries are chosen because they are “typical” for the country classification in the sense that they are in the middle range regarding change in unemployment rate from 2007 to 2011. The figure clearly shows the diverging unemployment trends for the four categories. Ireland represents the ‘crisis’ group, where there is a rapid increase in overall unemployment rate from 2007 and onwards. There is increasing unemployment in Portugal as well, but the line is clearly much less steep for this ‘mild crisis’ country. Also visible in Fig. 1, is the ‘small increase’ in Sweden from 2008 and onwards.

The trend for Poland – the included ‘no crisis’ country – shows us the downside of using only two cross-sections. When comparing 2007 and 2011, it appears that there has not been any changes in Polish labor demand at all: the unemployment rate is 9.6 and 9.7 percent respectively. Unfortunately, this hides the fact that the unemployment rate continued to decline in Poland, and from 2008 to 2010 there was actually a noticeable increase in the unemployment rate (from 7.1 to 9.7 percent). However, this is not an important problem for our purpose because we are mainly interested in what kind of “crisis case” the countries represent. In the following, we will investigate the unemployment risk for people with ill health in differing economic conditions, and try to see whether there are some patterns according to the severity of the crisis. We should nevertheless keep in mind that the current empirical strategy will only provide two “snapshots”, and some intra-country nuances will therefore be lost.

### Method and data

#### Data material

The cross-sectional part of the European Union Statistics on Income and Living Conditions (EU-SILC) data material is used in this paper. EU-SILC is an annual survey that covers all EU member countries, and Norway and Iceland. EU-SILC provides information on a wide range of variables, including health, employment status and basic demographics. Furthermore, the data material is harmonized cross-nationally for comparative purposes, and is therefore very well suited for our objective.

The EU-SILC consist of one cross-sectional and one longitudinal part, and the data are collected simultaneously. This means that (some of) the same individuals are included in both data sets, although it is only possible to localize these individuals in the panel. The EU-SILC panel is in a rotary format, where people are followed for a maximum of four years. In order for the same individuals not to contribute with several observations and hence biasing the results, we need a four-year gap between the pre-crisis and the crisis year. 2007 is chosen as *pre-crisis year* because the unemployment rate in Europe started to rise in 2008 (Eurostat 2015). Four years ahead – 2011 – is thus our *crisis year*. An alternative to using two (non-overlapping) cross-sections is to use the panel data (e.g. from 2008 to 2011), where it is possible to adjust for the fact that some people are contributing with several observations. However, to use the panel information is far from ideal because of attrition, which makes the samples less representative.

In the following, cross-sectional data from the years 2007 (pre-crisis) and 2011 (crisis) will be investigated in order to see whether the relationship between health and unemployment changes when the economy takes a turn for the worse. EU-SILC data is not available for the year 2007 for Croatia, Malta and Switzerland, and the total number of countries included in this study is therefore 28. There is no age restriction in the samples, but we include age dummies to adjust for possible cross-national differences in age composition.

#### Operationalization

Respondents who state to be unemployed on a question regarding their current economic status are coded 1 (else = 0) on the dummy variable *unemployment*. As a sensitivity test, the dependent variable is changed more in accordance with the International Labor Organizations’ (ILO) definition in all regressions. The dummy variable *ILO unemployment* consists of answers to two questions: “Actively looking for a job in the previous four weeks?” and “Available for work in the next two weeks?” Respondents answering yes on both is coded 1, otherwise 0. Individual-level unemployment is an outcome measure that is affected by a whole range of (unobserved) variables, both on the individual (e.g. educational credentials), regional (e.g. local labor market demand) and national (e.g. active labor market policies) level. Hence, the unemployment experience will most likely vary considerably across Europe due to differences in existing labor market institutions and/ or political solutions to economic downturns (austerity measures, for instance). Because of this cross-national unobserved heterogeneity, we have chosen to run all of the following analyses split by country.

*Limiting longstanding illness (LLSI)* is the most important independent variable in this paper. It is computed from answers to two questions: whether the respondent suffers from a chronic longstanding illness, and whether the respondent is limited in activities people usually do because of this. Those answering yes on both questions are coded 1 (else = 0). LLSI is preferred because it does not fluctuate as extensively cross-nationally as the self-rated general health (SRH) measure does. LLSI is hence more suitable from a comparative perspective. Nevertheless, all of the regressions have been performed with SRH as well, in order to check the robustness of the results. People reporting to have fair, bad, or very bad health is coded 1 (good or very good health = 0) on the *bad/fair health* dummy variable. Those with fair health are included because the number of people reporting bad or very bad health is low for some countries (e.g. approximately 5 percent in Sweden), yielding problems with statistical power.

A number of covariates is also included. Educational level consists of two dummy variables computed from a question on highest education attained. Pre-primary, primary and lower secondary is collapsed into *primary education*, while (upper) secondary and post-secondary non-tertiary is collapsed into *secondary education*. Higher educational qualifications is thus the reference category. Age is derived from questions on birth year and survey year, and thereafter recoded into five dummy variables: 16–25, 26–35, 46–55, 56–65, and above 65 years. Age 36–45 is the reference category. Married individuals could possibly be different on a range of unobserved characteristics, and a dummy denoting 1 for *married* (else = 0) corrects for this. Lastly, it is a well-known fact that women tend to report more ill health than men do [27]. A dichotomous variable for *women* (0 = men) is included in the regressions to adjust for this tendency.

#### Descriptive statistics

The number of observations for each of the 28 included countries in both survey years is presented in Table 7 in the appendix. The sample size fluctuates from 2869 in Iceland to 43666 in Italy. This implies that the models will be estimated with more precision for some of the countries, but all samples should be representative for the national population in question.

Table 2 presents descriptive statistics for the main dependent (*unemployment*) and independent (*LLSI*) variable, along with the proportions who report having *higher education* and being *woman*, split by survey year. Full descriptive statistics are not shown in order to save space, but are available on request. For all six ‘crisis countries’, there is roughly a doubling of the unemployment experience being reported from 2007 to 2011. The unemployment descriptives fit the country classification for the ‘minor crisis’ group as well, where there is a noticeable increase from 2007 to 2011. The ‘small increase’ group also corresponds well with the classification, with the possible exception of France, where there is almost no change (from 5.23 to 5.42), and Italy, where there is a noticeable increase (from 4.88 to 7.04). Lastly, there is not much change in the ‘no crisis’ group, and the most striking change is for the *better* (e.g. from 7.71 to 6.51 in Poland).

**Table 2:** Descriptive statistics on selected variables, by survey year (percentage)

	Unemployment		LLSI		Higher education		Woman	
	2007	2011	2007	2011	2007	2011	2007	2011
Crisis								
Estonia	3.08	7.48	32.79	33.70	20.09	26.17	53.93	58.74
Greece	5.01	11.53	17.39	22.27	15.66	17.12	51.95	52.04
Ireland	3.68	10.26	21.00	17.67	21.92	31.95	52.80	52.51
Latvia	4.42	11.76	31.17	32.87	16.64	20.90	57.67	57.40
Lithuania	3.83	8.56	26.46	24.35	20.58	23.68	54.25	56.93
Spain	6.18	11.96	16.66	18.55	20.96	23.09	52.40	52.27
Mild crisis								
Bulgaria	14.76	10.28	4.66	18.27	14.71	18.25	52.54	52.95
Cyprus	2.53	5.11	20.00	22.94	22.17	24.18	52.34	52.97
Denmark	2.30	3.57	15.13	16.50	26.63	30.74	51.54	52.01
Hungary	4.84	7.64	30.69	25.69	15.23	16.93	54.81	54.94
Iceland	0.84	4.36	12.30	17.27	21.47	24.59	49.29	51.68
Portugal	5.47	7.70	29.38	29.40	8.72	10.04	53.13	53.36
Slovenia	6.31	7.86	19.53	29.15	16.43	20.16	53.62	53.71
U.K.	1.71	3.09	22.43	23.32	22.35	31.47	53.68	52.67
Small increase								
Czech Republic	3.71	4.26	20.84	24.32	10.53	13.52	54.74	57.81
France	5.23	5.42	18.23	21.02	22.57	25.53	52.33	52.27
Italy	4.88	7.04	15.72	21.19	10.06	12.42	52.26	52.29
Slovakia	5.47	6.29	19.28	26.91	14.91	18.12	53.65	53.75
Sweden	2.73	3.87	19.08	16.16	27.32	29.82	51.32	52.36
No crisis								
Austria	3.22	3.86	18.85	23.16	15.65	17.50	52.72	52.71
Belgium	6.28	6.19	17.05	18.27	29.75	32.55	51.59	51.68
Finland	5.13	5.96	23.79	25.89	31.17	32.67	51.76	49.62
Germany	5.56	4.36	23.04	24.78	35.05	34.22	53.02	51.95
Luxembourg	4.22	3.87	11.92	11.52	25.48	22.42	50.18	50.58
Netherlands	1.07	1.74	17.18	21.83	30.26	33.16	54.39	54.25
Norway	1.68	1.82	18.30	16.53	29.10	36.50	48.84	46.90
Poland	7.71	6.51	21.70	22.32	12.21	15.53	53.60	53.93
Romania	3.30	2.45	17.26	24.64	9.19	10.89	52.45	52.29

Notes

Descriptive statistics only shown for the dependent variable (*unemployment*) and the independent variable of main interest (*LLSI*), along with two selected covariates (*higher education* and *woman*)

Full descriptive statistics are available on request

The overall level of unemployment is considerably lower for some of the countries compared with the official Eurostat statistics, indicating that the samples – in a number of cases – probably are positively selected (i.e. the most vulnerable groups are not reached). This is something worth remembering while interpreting the results.

The amount of LLSI being reported varies from approximately 12 (Luxembourg) to 33 percent (Estonia), although some of this difference is related to the age composition of the different samples. Mean age fluctuates from 43.54 (Luxembourg, in 2007) to 52.68 (Czech Republic, in 2011), and age dummies is therefore included in the following regressions. It should be mentioned, however, that there is still considerable cross-national variations in LLSI when only people of prime age (30–59 years) are considered (e.g. Italy 8.77 vs. Estonia 26.71, in 2007). A number of former ‘Eastern bloc’ countries (Estonia, Latvia, Lithuania, and Hungary) alongside Portugal tend to report the highest prevalence of limiting longstanding illness. In contrast, several Nordic countries (Finland excluded) and the Benelux- countries report comparatively low levels of LLSI.

Table 2 clearly shows the large differences in educational qualifications that exists across Europe, ranging from 8.72 percent in Portugal (2007) to 36.5 percent in Norway (2011) who hold higher education. Educational dummies is hence included in the regressions. There are no major gender skewness in either of the included samples. The gender skewness is largest in Estonia in 2011, where 58.74 percent are female. Lastly, it should be noted that the descriptive statistics do not make much sense for Bulgaria, where the unemployment prevalence *decreases* in the sample when the economy turned worse. There is apparently something wrong with the LLSI variable as well (very low level in 2007), and the data are clearly not to be trusted in the Bulgarian case.

#### Analysis

Linear probability models (OLS) are used throughout this paper. Although the dependent variable is a dummy (being unemployed or not), logistic regression is not preferred due to difficulties in the comparison of different samples and model specification [28, 29]. Nevertheless, logistic regression analysis has been performed as well in order to check the robustness of the results. The unemployment variable is regressed on ill health, with controls for marital status, educational qualifications, age and gender, yielding the following equation:

$$ Unemployed = {\beta}_1 Ill\  health + {\beta}_2 Married + {\beta}_3 Education + {\beta}_4 Age + {\beta}_5 Woman + \varepsilon $$

First, the regressions are run separately for the years 2007 and 2011, in order to compare the health coefficients. Afterwards, the data are pooled, and the same models are run along with a dummy variable for crisis year and an interaction term between 2011 and ill health:

$$ \begin{array}{l} Unemployed = {\beta}_1 Ill\  health + {\beta}_2 Married + {\beta}_3 Education + {\beta}_4 Age + {\beta}_5 Woman + \\ {}{B}_6 Crisis\  year + {\beta}_7 Crisis\  year\ *\  Ill\  health + \varepsilon \end{array} $$

These models will help us answering whether people with ill health have experienced unemployment to a lesser extent (than those with good health status) in the midst of an economic crisis (2011), compared with a pre-crisis period (2007). Next, a number of sensitivity tests are performed. Both the dependent and independent variable are changed, and logistic regression analysis is run in order to see whether the empirical pattern holds. In the last part of the analysis section, some descriptive statistics are presented, in order to further investigate potential changes in the composition of the unemployment population: (i) The unemployment prevalence among people reporting good and ill health in 2007 and 2011, and (ii) the share of unemployed people stating to have ill health in 2007 and 2011. All of the following analyses are split by country, since we are both interested in cross-national differences, and whether the results fit our crisis classification or not.

## Results

### Health and unemployment in diverging economic conditions

Table 3 reports results from an OLS regression of unemployment, by LLSI and a number of covariates (education, age, married and woman). The left column reports results for 2007, the right for 2011. Only the health coefficient is shown, since this is our prime interest. The results are *strikingly similar* for almost all of the 28 countries in Table 3. In merely three countries (Lithuania, Spain and Hungary) does the health coefficient change substantially from pre-crisis to crisis year.

**Table 3:** Results from OLS regression of unemployment, by LLSI and covariates

	2007	2011
A. Crisis		
Estonia	0.001 (0.004)	0.001 (0.007)
Greece	0.013** (0.006)	0.004 (0.008)
Ireland	0.007 (0.005)	−0.011 (0.009)
Latvia	0.008 (0.005)	0.007 (0.007)
Lithuania	0.000 (0.005)	−0.018** (0.007)
Spain	0.015*** (0.004)	−0.006 (0.005)
B. Mild crisis		
Bulgaria	−0.037** (0.017)	−0.013** (0.007)
Cyprus	0.003 (0.005)	0.004 (0.006)
Denmark	0.026*** (0.006)	0.020** (0.007)
Hungary	−0.010** (0.004)	−0.000 (0.004)
Iceland	−0.002 (0.005)	−0.005 (0.010)
Portugal	0.008 (0.006)	0.017** (0.006)
Slovenia	0.045*** (0.007)	0.037*** (0.007)
U.K.	0.003 (0.003)	0.005 (0.003)
C. Small increase		
Czech Republic	0.021*** (0.004)	0.017*** (0.004)
France	0.018*** (0.004)	0.015*** (0.004)
Italy	0.004 (0.003)	0.011** (0.003)
Slovakia	0.009 (0.006)	0.009 (0.005)
Sweden	0.016** (0.005)	0.031*** (0.006)
D. No crisis		
Austria	0.021*** (0.004)	0.040*** (0.004)
Belgium	0.035*** (0.006)	0.038*** (0.006)
Finland	0.012** (0.006)	0.014** (0.006)
Germany	0.034*** (0.004)	0.039*** (0.003)
Luxembourg	0.026*** (0.007)	0.037*** (0.006)
Netherlands	0.010*** (0.003)	0.010** (0.003)
Norway	0.003 (0.004)	0.015** (0.005)
Poland	−0.007* (0.004)	−0.007* (0.004)
Romania	−0.001 (0.004)	−0.002 (0.003)

Significance level

*** = 0.01 ** = 0.05 * = 0.1 NS/(empty) = > 0.1

Covariates

Gender dummy, marital status dummy, two educational level dummies, and five age dummies (ref.: 36–45 years)

Only LLSI coefficients shown. Full models available on request

In several cases, the health coefficient is almost identical for the two survey years, for instance in Estonia (0.001 and 0.001), the U.K (0.003 and 0.005), Slovakia (0.009 and 0.009) and the Netherlands (0.010 and 0.010). In Portugal, Italy and Norway, there is a slightly higher effect size in 2011 causing the coefficient to become statistically significant. In general, however, the effect size is quite small and often far from significant. This means that in many European countries, there is no major unemployment disadvantage for people with ill health, once education, age, marital status and gender is accounted for. It should nevertheless be noted that there exists a heightened unemployment likelihood for people with LLSI in a number of countries, including Denmark, Slovenia, Austria, Belgium, Germany and Luxembourg.

Table 4 investigates a related question, namely whether people with ill health are unemployed to a lesser extent in the crisis year, when unemployment becomes more widespread across Europe. In other words, has people without health problems experienced the main bulk of the unemployment incidences? The data for 2007 and 2011 are now pooled. The regression is similar as before, except for the inclusion of a dummy variable for crisis year (coefficients shown in left column) and an interaction term between crisis year and LLSI (coefficients shown in right column). The year dummies indicate the extent to which unemployment probabilities have changed for people with good health. The interaction terms, on the other hand, will tell us whether respondents with ill health have a different unemployment likelihood in 2011, compared with 2007.

**Table 4:** Pooled sample: Results from OLS regression of unemployment, by LLSI, 2011, LLSI x 2011, and covariates

	2011	LLSI x 2011
A. Crisis		
Estonia	0.057*** (0.004)	−0.023*** (0.006)
Greece	0.084*** (0.004)	−0.067*** (0.008)
Ireland	0.072*** (0.004)	−0.036*** (0.009)
Latvia	0.090*** (0.004)	−0.042*** (0.008)
Lithuania	0.059*** (0.004)	−0.042*** (0.008)
Spain	0.071*** (0.003)	−0.049*** (0.006)
B. Mild crisis		
Bulgaria	−0.029*** (0.004)	0.039*** (0.017)
Cyprus	0.029*** (0.003)	−0.010 (0.007)
Denmark	0.016*** (0.003)	−0.012 (0.009)
Hungary	0.028*** (0.003)	0.000 (0.005)
Iceland	0.038*** (0.004)	−0.009 (0.012)
Portugal	0.029*** (0.004)	−0.009 (0.007)
Slovenia	0.021*** (0.004)	−0.018** (0.009)
U.K.	0.015*** (0.002)	−0.002 (0.004)
C. Small increase		
Czech Republic	0.012*** (0.003)	−0.008 (0.005)
France	0.005** (0.002)	−0.010* (0.006)
Italy	0.025*** (0.002)	−0.006 (0.004)
Slovakia	0.011** (0.003)	−0.005 (0.007)
Sweden	0.012*** (0.003)	0.012 (0.008)
D. No crisis		
Austria	0.002 (0.003)	0.017** (0.006)
Belgium	−0.003 (0.003)	−0.003 (0.008)
Finland	0.012** (0.004)	−0.002 (0.008)
Germany	−0.016*** (0.002)	0.008 (0.004)
Luxembourg	−0.002 (0.003)	0.010 (0.009)
Netherlands	0.008*** (0.002)	−0.003 (0.004)
Norway	0.002 (0.003)	0.012* (0.007)
Poland	−0.007** (0.002)	0.003 (0.005)
Romania	−0.007** (0.002)	0.005 (0.005)

Significance level

*** = 0.01 ** = 0.05 * = 0.1 NS/(empty) = > 0.1

Covariates

Gender dummy, marital status dummy, two educational level dummies, and five age dummies (ref.: 36–45 years)

Only the coefficients for 2011 and the interaction term LLSI × 2011 is shown. Full models available on request

The 2011 dummy is, naturally, both large and highly significant in all the ‘crisis’ countries, with an especially large effect size in Latvia (0.090) and Greece (0.084). The crisis dummy is smaller (but still significant) for the ‘minor crisis’ countries and in the ‘small increase’ group as well. In the ‘no crisis’ countries, the year dummy is positive and significant in Finland and the Netherlands (small coefficient in both cases), and significantly *negative* in three countries (Germany, Poland and Romania). Our prime interest, however, is the interaction terms, which show a distinct pattern.

For all of the ‘crisis’ countries, the interaction term is negative and statistically significant, but this is almost never the case for the remaining 22 countries (the exceptions being Slovenia and France). It is worth noting that the interaction terms are often negative in the ‘mild crisis’ and ‘small increase’ group as well, but they are considerably smaller in effect size and fail to reach statistical significance. This shows that both a high *level* and a rapid *increase* in the unemployment rate seems to be necessary in order for people with good health status to become overrepresented in the unemployment population.

#### Robustness checks

The results presented thus far points to two main findings. First, the unemployment risk for people with ill health is a very stable phenomenon, in the sense that the LLSI coefficient is remarkably similar in 2007 and 2011 for almost all of the 28 European countries. Second, people with good health status has experienced the main bulk of the unemployment incidences during the crisis, but only in countries with both a high and increasing overall unemployment level. However, these results might be sensitive to the choice of independent and dependent variable, and to the choice of linear instead of logistic regression.

The pattern of similarity over time in people with ill health’s unemployment probabilities, compared with people with good health, also holds when the independent variable is changed to *bad/fair health* (see Table 8 in appendix). Iceland is the only country where the health coefficient changes somewhat from 2007 to 2011. This is also the case when the dependent variable is changed to *ILO unemployment*, where Spain is the country with most apparent change (see Table 9 in appendix). There is some minor changes in Belgium, Denmark, Finland and Norway as well, but the main finding is still stability over time.

All the regressions have been rerun with a change in the dependent and independent variable on the pooled data as well. The main findings hold in both model specifications, except for a couple of slight differences. First, when *bad/fair health* is used instead of LLSI (see Table 10), the effect size of the interaction term is lowered somewhat in all ‘crisis countries’, and the interaction is no longer significant in Ireland (b = −0.014, SE = 0.009). Second, the interaction term is now negative and statistically significant on the five percent level for Belgium (b = −0.015), Cyprus (b = −0.013), Iceland (b = −0.024) and the Czech Republic (b = −0.010) as well. Third, when the dependent variable is switched to *ILO unemployment* (see Table 11), the interaction term is negative and significant on the five percent level for Hungary, Italy and the Czech Republic, but the effect sizes are all rather small (between −0.011 and −0.014). These minor inconsistencies do not, however, change the overarching conclusion: that the unemployment likelihood is lowered substantially for people with health issues in countries hit hard by the recession.

The preceding analysis have also been calculated using logistic regression (see Tables 12 and 13). It should be stressed that it is challenging to compare results across different samples using logistic regression, because the variance is fixed (at 3.29) in the logistic distribution causing more problems with unobserved heterogeneity in the model specification [28, 29]. However, if the main empirical pattern derived from the linear models is found using logistic regression analysis as well, we can be more confident in the presented findings. This definitely seems to be the case for both the analysis split by survey year (Table 12) and the analysis of the pooled data (Table 13). Regarding the former, there are few noticeable changes from 2007 to 2011 (main exceptions: Greece, Spain, Hungary and Norway). For the latter, there is still a lower unemployment likelihood for people with ill health in the ‘crisis countries’, although the interaction term fails to reach statistical significance for both Estonia and Latvia. Furthermore, it should be noted that the interaction term is negative and significant for Denmark and Slovenia as well. To summarize, the choice of linear over logistic regression analysis does not seem to be responsible for the presented findings.

#### Compositional changes: descriptive evidence

Lastly, we turn to some descriptive evidence on the compositional changes of the unemployment population. For brevity, only the results for the main dependent and independent variable are presented. The main reason for people with ill health’s unemployment probability being lower in 2011 in the ‘crisis countries’ is shown in Table 5, where the percentages of people with good health (left columns) and LLSI (right columns) who report to be unemployed is shown for the two survey years. Clearly, the differences between 2007 and 2011 are larger in the good health group than in the LLSI group. In Latvia, for instance, the increase in unemployment prevalence is much larger among those reporting good health (from 4.83 to 13.65) than among those with LLSI (from 3.53 to 7.91). This pattern holds for all six ‘crisis countries’, and is most evident in Spain and Greece. The difference between people with good health and people reporting LLSI is – as shown in Table 4 above – statistically significant on the 99 percent level for all the ‘crisis countries’. People with LLSI have experienced significantly less of the unemployment increase in Slovenia as well, whereas the opposite is the case in Austria and Norway. The latter result is easy to notice in Table 5, where it is only among people reporting LLSI there is a significant increase in unemployment prevalence (e.g. Austria: from 3.04 to 3.22 for good health, and from 4.00 to 5.98 for LLSI).

**Table 5:** Unemployment prevalence in 2007 and 2011 among people with good health (1) and LLSI (2) (percent)

	(1) Good health		(2) LLSI	
A. Crisis	2007	2011	2007	2011
Estonia	3.45	8.56***	2.34	5.36***
Greece	5.44	13.61***	2.98	4.26**
Ireland	3.80	10.97***	3.24	6.98***
Latvia	4.83	13.65***	3.53	7.91***
Lithuania	4.20	9.92***	2.81	4.33***
Spain	6.37	13.12***	5.26	6.85***
B. Mild crisis	2007	2011	2007	2011
Bulgaria	15.28	11.30***	4.17	4.25
Cyprus	2.75	5.67***	1.66	3.22**
Denmark	1.90	3.31***	4.57	4.90
Hungary	5.81	8.44***	2.64	5.30***
Iceland	0.87	4.55***	0.57	3.47***
Portugal	6.05	8.63***	4.07	5.47***
Slovenia	5.84	7.73***	8.24	8.17
U.K.	1.78	3.22***	1.48	2.69***
C. Small increase				
Czech Republic	3.67	4.39**	3.86	3.86
France	5.27	5.56	5.07	4.90
Italy	5.34	7.77***	2.40	4.34***
Slovakia	5.84	6.93**	3.93	4.56
Sweden	2.55	3.53**	3.48	5.63**
D. No crisis	2007	2011	2007	2011
Austria	3.04	3.22	4.00	5.98**
Belgium	5.84	5.73	8.40	8.26
Finland	4.97	5.84**	5.66	6.30
Germany	5.05	3.69***	7.28	6.43*
Luxembourg	4.08	3.56*	5.30	6.22
Netherlands	0.88	1.60***	2.00	2.23
Norway	1.67	1.59	1.73	3.02*
Poland	8.66	7.35***	4.26	3.58**
Romania	3.73	2.98***	1.26	0.81*

Notes

*T*-test on the difference between 2007 and 2011

Significance levels: *** = 0.01 ** = 0.05 * = 0.1 NS/(empty) = > 0.1

Further evidence of the changing health composition is presented in Table 6, which shows the share of unemployed people stating to have LLSI. For all six ‘crisis countries’, the share of people reporting health problems among the unemployed is lower in 2011 than in 2007 (only significantly so in Ireland, Lithuania and Spain). Remember, however, that these are the “raw” and unadjusted differences, and the number of observations are much more limited when the data are structured in this manner (e.g. Estonia: N = 366 and 642). The statistical uncertainty is therefore a more pressing issue. For the 22 remaining countries there tends to be *more* people with ill health in the unemployment population in the crisis year, although these upward changes are only significant in Austria, Germany and Italy (Slovenia on the ten percent level). Denmark, Portugal, Sweden and the Netherlands are the exceptions, where there are slightly less (but never significantly so) people with LLSI among the unemployed in 2011.

**Table 6:** Share of the unemployed stating to have LLSI in 2007 and 2011 (percent)

	2007	2011
A. Crisis		
Estonia	24.86 (N = 366)	24.14 (N = 642)
Greece	10.34 (N = 619)	8.24 (N = 1457)
Ireland	18.45 (N = 401)	12.01*** (N = 841)
Latvia	24.88 (N = 410)	22.10 (N = 1575)
Lithuania	19.42 (N = 417)	12.31*** (N = 804)
Spain	14.16 (N = 1773)	10.63*** (N = 3461)
B. Mild crisis		
Bulgaria	1.32 (N = 1367)	5.97*** (N = 1575)
Cyprus	13.08 (N = 214)	14.43 (N = 485)
Denmark	30.08 (N = 133)	22.63 (N = 190)
Hungary	16.74 (N = 890)	17.83 (N = 1879)
Iceland	8.33 (N = 24)	13.74 (N = 131)
Portugal	21.88 (N = 544)	20.89 (N = 962)
Slovenia	25.50 (N = 549)	30.30* (N = 726)
U.K.	19.41 (N = 273)	20.26 (N = 454)
C. Small increase		
Czech Republic	21.68 (N = 655)	22.05 (N = 567)
France	17.67 (N = 1058)	18.99 (N = 1153)
Italy	7.74 (N = 2132)	13.05*** (N = 2750)
Slovakia	13.85 (N = 686)	19.52*** (N = 835)
Sweden	24.34 (N = 189)	23.55 (N = 259)
D. No crisis		
Austria	23.43 (N = 431)	35.89*** (N = 443)
Belgium	22.79 (N = 768)	24.35 (N = 694)
Finland	26.23 (N = 469)	27.36 (N = 541)
Germany	30.17 (N = 1442)	36.49*** (N = 1055)
Luxembourg	14.97 (N = 334)	18.51 (N = 443)
Netherlands	32.11 (N = 109)	28.02 (N = 182)
Norway	18.81 (N = 101)	27.38 (N = 84)
Poland	11.99 (N = 2528)	12.26 (N = 1843)
Romania	6.57 (N = 563)	8.18 (N = 391)

Notes

*T*-test on the difference between 2007 and 2011

Significance levels: *** = 0.01 ** = 0.05 * = 0.1 NS/(empty) = > 0.1

Number of observations in parentheses

To summarize, people with ill health’s unemployment likelihood, compared with people reporting good health, is *remarkably stable* over time in Europe, and there is no evidence of the relationship being modified by a sudden increase in the unemployment rate. However, a different empirical pattern emerges when we pool the data for 2007 and 2011, and investigate the interplay between ill health and crisis year. People with ill health have a *lower* unemployment probability in the crisis year, but only in countries hit hard by the recession as indicated by a high and rising unemployment level. This result is mainly due to *compositional changes* on health characteristics in the unemployment population, as people reporting good health have experienced unemployment to a higher extent than those with ill health in the ‘crisis countries’. In the following and last section, the presented results will be discussed in greater detail.

## Discussion

Before we turn to a discussion of the findings, a number of important shortcomings should be mentioned. The empirical strategy in this paper only provides us with “snapshots”, and we are not able to say to what degree the presented statistical associations are of a *causal* nature (i.e. that people lose their jobs because of bad health status). Similarly, the naïve regression approach chosen cannot help us teasing out the extent to which the relationship between ill health and unemployment likelihood is driven by selective processes, health effects of unemployment, and/ or omitted variable bias (e.g. personality characteristics, cognitive abilities, etc.). It is highly likely, however, that the main bulk of the changing association between health and unemployment likelihood in the ‘crisis countries’ is due to selective processes, for two reasons. Firstly, because of the large numbers of unemployment episodes, which probably outnumber health declines due to unemployment. Secondly, there is no general trend towards more ill health being reported in 2011 among the ‘crisis countries’ (see Table 2), as one would expect if people deteriorate in health because of the unemployment experience.

Furthermore, the data material is not detailed enough to disentangle to what extent the unemployment prevalence is of a short- or a long-term kind, and whether there are health differentials in the length of the unemployment spell. It might be the case, for instance, that people with ill health are overrepresented among the long-term unemployed, because they have trouble in accessing the labor market [11–13]. This could, in fact, be a particularly pressing issue in the ‘crisis countries’, where the demand for labor has been continuingly low in the years 2008–2011. This means that employers can “skim the cream” to a higher extent in recruitment processes, and all negative productivity signals (e.g. bad health status, previous unemployment episodes, old age) attached to an applicant will most likely lead to a lower hiring probability. Consequently, even though people with ill health have experienced the rise in unemployment to a lower extent overall than people with good health in the ‘crisis countries’ , they could still be overrepresented among those who are more permanently disadvantaged on the labor market (i.e. the long-term unemployed).

There is some evidence indicating that vulnerable groups are underrepresented in (a number of) the EU-SILC samples. When comparing the official Eurostat unemployment statistics with the reported unemployment in EU-SILC, there were some noticeable differences. In Ireland, the reported amount of unemployment is 3.7 and 10.26 for the years 2007 and 2011 respectively, while the official statistics was 4.7 and 14.7. This could be due to *underreporting*, i.e. respondents (wrongly) classifying themselves as something other than unemployed. If people with ill health do this to a higher extent than those with good health, the presented results could be biased. There is, however, no reason to suspect that this tendency should be much stronger in the ‘crisis countries’, and the main findings of this study are probably not driven by such processes. Additionally, it is possible that those not reached in the surveys (and/ or the non-response group) has a high probability of both being unemployed and having health problems, which would bias the estimates. Yet, given the fact that – for most countries – between 20 and 30 percent report to have a limiting longstanding illness, it seems unlikely that people with health issues are severely underrepresented in the sample.

This study has investigated the following research question: *Do people with ill health experience unemployment to a lesser extent than those with good health during the economic downturn in Europe?* The answer is yes, but only in countries in which there is both a high and rapidly growing unemployment rate. This means that the overall *health composition* has changed for the healthier in the countries classified as experiencing a full-blown crisis. In the remaining countries, in contrast, the unemployment prevalence for people with LLSI have – if anything – increased. Thus, evidence from 28 European countries indicates that less severe economic downturns will probably not change the health composition of the unemployment population at all, only a severe crisis will.

The remaining question is how to explain this empirical pattern? That people with ill health are selected for unemployment in a crisis of minor or intermediate level is no surprise, and there are at least four reasons to expect this. Firstly, health status might function as a *productivity proxy*, and employers might therefore be reluctant to hire (and more inclined to fire) those with ill health. Secondly, because people with ill health often have troubles in accessing the labor market they will have *less seniority* [30, 31], and therefore a higher lay-off risk. Thirdly, the problems in gaining employment for those with health troubles could be due to *scarring effects of unemployment* [32, 33]. Hence, employers might be indifferent to the health status per se, but rather be skeptical about the accumulated unemployment on the CV, yielding lower hiring probability and less seniority. Fourthly and lastly, some employers might even have *discriminatory preferences* [34, 35] against those with health problems, possibly causing both difficulties in gaining employment and a higher unemployment likelihood.

These processes are, however, not as important during severe recessions, when unemployment becomes a mass phenomenon. In this situation, employers have to make large numbers of employees redundant (e.g. when an entire factory closes down), and there will naturally be less selectivity on both health- and other characteristics. And because having good health is more common than having health problems, the unemployment population will inevitably take a compositional change for the healthier.

Another important question is how the findings from this paper corresponds to the existing literature on health and unemployment. Our results might seem to contradict those of a recent study also employing the EU-SILC, which finds that people with health limitations were *more* prone to unemployment in Europe [36]. However, the study uses longitudinal data (with accompanying attrition difficulties) and the sample is limited to people employed at the start of the observational window, making the comparison of results with the current study very challenging.

More in line with our empirical strategy are two studies of unemployment and mortality rates from Finland, who experienced a severe economic crisis in the 1990’s. The unemployment rate was approximately 5 % until 1989. By 1992, the unemployment rate was 15 %, and reached a peak of 19 % in 1994. Excess mortality of individuals who experienced unemployment before the rise in unemployment was greater than for individuals experiencing unemployment during the recession [37]. Similarly, a more recent Finnish study found that the mortality hazard of the unemployed were considerably higher during the more favorable economic climate, and the association between mortality and unemployment were weaker among workers in strongly downsizing firms [38]. Correspondingly, findings from Australia indicate that young unemployed peoples’ health is worse when the unemployment rate is low, compared to when the unemployment rate is high [39].

These findings fit well with our results, showing that the unemployed are “healthier” on average in European countries where the unemployment rate is both high and rising. In other words, the unemployment population is *positively selected* on health characteristics in ‘crisis countries’, something which probably is able to explain the less serious health effects of unemployment found in the three above-mentioned studies. The main alternative explanation can be termed “*the more, the merrier*”. It is possible that there is less psychosocial stress and stigma associated with being unemployed when redundancies are more widespread, and that the negative health consequences therefore are muted. Although this could be a key factor in some cases, it is probably much less important than the explanation emphasizing that the unemployment population is positively selected on health. Results from the present paper highlights the importance of such selective processes, and how these are related to the severity of the economic crisis. Future comparative research – preferably using individual level longitudinal data with a longer time span than the EU-SILC – should investigate whether the health effects of unemployment are less prominent in countries where unemployment became a mass phenomenon during “the Great Recession”.

## Conclusion

There has to be a rather sever economic downturn in order for the health composition of the unemployment population to change significantly. In countries with a high and increasing overall unemployment rate, people with ill health experience unemployment to a *lower* extent than people with good health. This tendency is not observed for countries in which there is a “milder” crisis. If anything, people with ill health seems to be *more* prone to unemployment in countries where the crisis impact is on a small or intermediate level. This could indicate that people with LLSI are among the first to be laid off when the economy takes a turn for the worse. However, only when there is a full-blown economic crisis – with a high and rapidly increasing unemployment level – will the unemployment composition change for the better in health terms.

## Appendix

**Table 7:** Number of observations for 28 European countries in 2007 and 2011

Country	2007	2011
Austria	13382	11471
Belgium	12234	11203
Bulgaria	9261	15324
Cyprus	8453	9491
Czech Republic	17666	13309
Denmark	5782	5322
Estonia	11872	8585
Finland	9138	9073
France	20215	21260
Germany	25932	24170
Greece	12346	12641
Hungary	18403	24609
Iceland	2869	3005
Ireland	10885	8196
Italy	43666	39062
Latvia	9266	13388
Lithuania	10885	9397
Luxembourg	7908	11448
Netherlands	10211	10465
Norway	6001	4603
Poland	32798	28304
Portugal	9942	12488
Romania	17042	15974
Slovakia	12533	13271
Slovenia	8701	9238
Spain	28652	28941
Sweden	6925	6700
United Kingdom	15972	14670

Notes

Only participants who answered the health questions are included in the sample

Individuals with missing information on health variables were dropped

*2007 EU-SILC- data not available for Croatia, Malta and Switzerland*

**Table 8:** Sensitivity test: Results from OLS regression of unemployment, by bad/fair health and covariates

	2007	2011
A. Crisis		
Estonia	0.015*** (0.004)	0.030*** (0.007)
Greece	0.012** (0.005)	0.016** (0.007)
Ireland	0.004 (0.005)	0.009 (0.009)
Latvia	0.022*** (0.005)	0.032*** (0.007)
Lithuania	0.012** (0.005)	0.029*** (0.007)
Spain	0.021*** (0.003)	0.014** (0.005)
B. Mild crisis		
Bulgaria	0.033** (0.009)	0.024*** (0.006)
Cyprus	0.010** (0.005)	0.009 (0.006)
Denmark	0.024*** (0.005)	0.027*** (0.006)
Hungary	0.012** (0.004)	0.038*** (0.004)
Iceland	0.016*** (0.004)	0.001 (0.009)
Portugal	0.017** (0.006)	0.030*** (0.006)
Slovenia	0.034*** (0.006)	0.047*** (0.007)
U.K.	0.005* (0.003)	0.010** (0.003)
C. Small increase		
Czech Republic	0.030*** (0.004)	0.022*** (0.004)
France	0.018*** (0.004)	0.018*** (0.004)
Italy	0.013*** (0.002)	0.024*** (0.003)
Slovakia	0.013** (0.005)	0.025*** (0.005)
Sweden	0.023*** (0.005)	0.031*** (0.006)
D. No crisis		
Austria	0.040*** (0.004)	0.051*** (0.004)
Belgium	0.053*** (0.005)	0.042*** (0.006)
Finland	0.031*** (0.006)	0.033*** (0.006)
Germany	0.045*** (0.003)	0.045*** (0.003)
Luxembourg	0.034*** (0.006)	0.031*** (0.004)
Norway	0.010** (0.004)	0.018*** (0.005)
Netherlands	0.009*** (0.003)	0.010** (0.003)
Poland	0.008** (0.004)	0.016*** (0.004)
Romania	0.003 (0.004)	0.002 (0.003)

Significance level

*** = 0.01 ** = 0.05 * = 0.1 NS/(empty) = > 0.1

Covariates

Gender dummy, marital status dummy, two educational level dummies, and five age dummies (ref.: 36–45 years)

Only bad/fair health coefficients shown. Full models available on request

**Table 9:** Sensitivity test: Results from OLS regression of ILO unemployment, by LLSI and covariates

	2007	2011
A. Crisis		
Estonia	0.002 (0.004)	0.005 (0.007)
Greece	0.005 (0.005)	-0.000 (0.007)
Ireland	-0.010** (0.004)	-0.036*** (0.009)
Latvia	0.007 (0.005)	0.018** (0.006)
Lithuania	-0.002 (0.004)	-0.010 (0.007)
Spain	0.010** (0.004)	-0.010** (0.005)
B. Mild crisis		
Bulgaria	-0.025** (0.012)	-0.004 (0.006)
Cyprus	0.004 (0.005)	0.007 (0.006)
Denmark	0.014** (0.005)	0.005 (0.007)
Hungary	-0.001 (0.004)	-0.003 (0.004)
Iceland	0.003 (0.007)	0.004 (0.011)
Portugal	-0.003 (0.005)	0.002 (0.005)
Slovenia	0.010** (0.005)	0.009* (0.005)
U.K.	0.003 (0.003)	0.008** (0.004)
C. Small increase		
Czech Republic	0.021*** (0.004)	0.015*** (0.004)
France	0.009** (0.004)	0.007* (0.004)
Italy	0.004 (0.003)	0.007** (0.003)
Slovakia	0.011** (0.005)	0.006 (0.005)
Sweden	-0.002 (0.005)	0.007 (0.006)
D. No crisis		
Austria	0.005 (0.004)	0.012** (0.004)
Belgium	0.010** (0.005)	-0.000 (0.005)
Finland	0.009* (0.005)	-0.001 (0.005)
Germany	0.016*** (0.003)	0.019*** (0.003)
Luxembourg	0.015** (0.007)	0.013** (0.005)
Netherlands	0.014*** (0.003)	0.006** (0.002)
Norway	0.003 (0.004)	0.018*** (0.005)
Poland	-0.007** (0.003)	-0.009** (0.003)
Romania	-0.004 (0.004)	-0.001 (0.003)

Significance level

*** = 0.01 ** = 0.05 * = 0.1 NS/(empty) = > 0.1

Covariates

Gender dummy, marital status dummy, two educational level dummies, and five age dummies (ref.: 36–45 years)

Only LLSI coefficients shown. Full models available on request

**Table 10:** Sensitivity test, pooled sample: Results from OLS regression of unemployment, by bad/fair health, 2011, bad/fair health × 2011, and covariates

	2011	Bad/fair health × 2011
A. Crisis		
Estonia	0.056*** (0.004)	-0.013** (0.006)
Greece	0.089*** (0.004)	-0.063*** (0.008)
Ireland	0.068*** (0.004)	-0.014 (0.009)
Latvia	0.097*** (0.006)	-0.033*** (0.008)
Lithuania	0.062*** (0.005)	-0.022** (0.007)
Spain	0.076*** (0.003)	-0.039*** (0.005)
B. Mild crisis		
Bulgaria	-0.031*** (0.005)	0.012 (0.008)
Cyprus	0.030*** (0.003)	-0.013* (0.007)
Denmark	0.014*** (0.004)	-0.003 (0.007)
Hungary	0.027*** (0.003)	0.008* (0.005)
Iceland	0.041*** (0.005)	-0.024** (0.010)
Portugal	0.030*** (0.005)	-0.006 (0.007)
Slovenia	0.021*** (0.005)	-0.000 (0.008)
U.K.	0.015*** (0.002)	0.001 (0.004)
C. Small increase		
Czech Republic	0.015*** (0.003)	-0.010** (0.004)
France	0.005* (0.003)	-0.007 (0.005)
Italy	0.026*** (0.002)	-0.003 (0.003)
Slovakia	0.010** (0.004)	0.004 (0.006)
Sweden	0.013*** (0.003)	0.005 (0.007)
D. No crisis		
Austria	0.002 (0.003)	0.011** (0.005)
Belgium	0.001 (0.004)	-0.015** (0.007)
Finland	0.012** (0.004)	-0.003 (0.007)
Germany	-0.012*** (0.002)	0.004 (0.004)
Luxembourg	-0.001 (0.003)	-0.003 (0.007)
Netherlands	0.008*** (0.002)	-0.003 (0.004)
Norway	0.001 (0.003)	0.009 (0.006)
Poland	-0.010*** (0.003)	0.008* (0.004)
Romania	-0.008*** (0.002)	0.006* (0.004)

Significance level

*** = 0.01 ** = 0.05 * = 0.1 NS/(empty) = > 0.1

Covariates

Gender dummy, marital status dummy, two educational level dummies, and five age dummies (Ref.: 36–45 years)

Only the coefficients for 2011 and the interaction term bad/fair health × 2011 is shown. Full models available on request

**Table 11:** Sensitivity test, pooled sample: Results from OLS regression of ILO unemployment, by LLSI, 2011, LLSI × 2011, and covariates

	2011	LLSI × 2011
A. Crisis		
Estonia	0.061*** (0.004)	-0.022** (0.006)
Greece	0.076*** (0.003)	-0.057*** (0.008)
Ireland	0.073*** (0.004)	-0.049*** (0.009)
Latvia	0.083*** (0.004)	-0.031*** (0.008)
Lithuania	0.058*** (0.004)	-0.031*** (0.007)
Spain	0.066*** (0.002)	-0.048*** (0.006)
B. Mild crisis		
Bulgaria	0.012*** (0.003)	0.013 (0.013)
Cyprus	0.029*** (0.003)	-0.013* (0.007)
Denmark	0.020*** (0.003)	-0.015* (0.009)
Hungary	0.031*** (0.003)	-0.011** (0.005)
Iceland	0.044*** (0.005)	-0.009 (0.014)
Portugal	0.023*** (0.004)	-0.010 (0.007)
Slovenia	0.016*** (0.003)	-0.009 (0.006)
U.K.	0.018*** (0.002)	-0.002 (0.004)
C. Small increase		
Czech Republic	0.010*** (0.002)	-0.011** (0.005)
France	0.007** (0.002)	-0.006 (0.005)
Italy	0.031*** (0.002)	-0.014*** (0.004)
Slovakia	0.015** (0.003)	-0.012* (0.007)
Sweden	0.011*** (0.003)	0.003 (0.008)
D. No crisis		
Austria	0.001 (0.002)	0.005 (0.005)
Belgium	0.004 (0.003)	-0.011* (0.006)
Finland	0.014*** (0.003)	-0.011* (0.006)
Germany	-0.014*** (0.002)	0.008** (0.004)
Luxembourg	-0.006** (0.003)	-0.001 (0.009)
Netherlands	-0.001 (0.002)	-0.007* (0.004)
Norway	-0.001 (0.003)	0.015** (0.007)
Poland	0.008*** (0.002)	-0.007 (0.004)
Romania	-0.005** (0.002)	0.006 (0.004)

Significance level

*** = 0.01 ** = 0.05 * = 0.1 NS/(empty) = > 0.1

Covariates

Gender dummy, marital status dummy, two educational level dummies, and five age dummies (Ref.: 36–45 years)

Only the coefficients for 2011 and the interaction term LLSI × 2011 is shown. Full models available on request

**Table 12:** Results from logistic regression of unemployment, by LLSI and covariates

	2007	2011
A. Crisis		
Estonia	1.000 (0.134)	1.008 (0.107)
Greece	1.708*** (0.258)	1.104 (0.122)
Ireland	1.281* (0.182)	0.864 (0.106)
Latvia	1.225 (0.154)	1.076 (0.077)
Lithuania	0.974 (0.133)	0.754** (0.090)
Spain	1.378*** (0.103)	0.908 (0.057)
B. Mild crisis		
Bulgaria	0.536** (0.141)	0.753** (0.089)
Cyprus	1.214 (0.265)	1.118 (0.161)
Denmark	2.436*** (0.495)	1.693** (0.316)
Hungary	0.774** (0.077)	1.020 (0.071)
Iceland	0.790 (0.596)	0.820 (0.220)
Portugal	1.188 (0.137)	1.327** (0.120)
Slovenia	2.167*** (0.243)	1.703*** (0.158)
U.K.	1.221 (0.200)	1.212 (0.153)
C. Small increase		
Czech Republic	2.030*** (0.214)	1.645*** (0.186)
France	1.495*** (0.132)	1.427*** (0.117)
Italy	1.253** (0.109)	1.314*** (0.082)
Slovakia	1.240* (0.150)	1.204* (0.117)
Sweden	1.812** (0.326)	2.273*** (0.365)
D. No crisis		
Austria	2.053*** (0.257)	2.791*** (0.310)
Belgium	1.767*** (0.170)	1.911*** (0.193)
Finland	1.274** (0.146)	1.299** (0.138)
Germany	1.867*** (0.121)	2.295*** (0.168)
Luxembourg	1.962** (0.329)	2.640*** (0.360)
Netherlands	2.130** (0.464)	1.740** (0.305)
Norway	1.225 (0.326)	2.185** (0.577)
Poland	0.880* (0.059)	0.859** (0.067)
Romania	0.945 (0.171)	0.830 (0.164)

Significance level

*** = 0.01 ** = 0.05 * = 0.1 NS/(empty) = > 0.1

Covariates

Gender dummy, marital status dummy, two educational level dummies, and five age dummies (Ref.: 36-45 years)

Only the odds ratio for LLSI is shown. Full models available on request

**Table 13:** Pooled sample: Results from logistic regression of unemployment, by LLSI, 2011, LLSI × 2011, and covariates

	2011	LLSI × 2011
A. Crisis		
Estonia	3.027*** (0.240)	0.930 (0.148)
Greece	2.978*** (0.161)	0.611** (0.106)
Ireland	3.397*** (0.243)	0.690** (0.123)
Latvia	3.302*** (0.222)	0.823 (0.113)
Lithuania	2.673*** (0.188)	0.670** (0.116)
Spain	2.388*** (0.080)	0.654*** (0.062)
B. Mild crisis		
Bulgaria	0.760*** (0.032)	1.348 (0.382)
Cyprus	2.104*** (0.190)	1.079 (0.266)
Denmark	1.944*** (0.264)	0.579** (0.154)
Hungary	1.528*** (0.072)	1.390** (0.157)
Iceland	5.708*** (1.348)	1.069 (0.842)
Portugal	1.539*** (0.098)	1.056 (0.146)
Slovenia	1.412*** (0.099)	0.743** (0.102)
U.K.	1.895*** (0.167)	1.020 (0.202)
C. Small increase		
Czech Republic	1.331*** (0.090)	0.862 (0.126)
France	1.121** (0.055)	0.856 (0.099)
Italy	1.545*** (0.050)	1.222* (0.127)
Slovakia	1.195** (0.071)	0.987 (0.146)
Sweden	1.545*** (0.174)	1.296 (0.302)
D. No crisis		
Austria	1.046 (0.087)	1.363* (0.216)
Belgium	0.951 (0.060)	0.989 (0.132)
Finland	1.244** (0.096)	0.970 (0.146)
Germany	0.657*** (0.034)	1.195* (0.111)
Luxembourg	0.934 (0.076)	1.233 (0.254)
Netherlands	1.872*** (0.275)	0.660 (0.177)
Norway	1.106 (0.191)	1.735 (0.627)
Poland	0.908** (0.031)	0.983 (0.096)
Romania	0.802** (0.057)	0.991 (0.254)

Significance level

*** = 0.01 ** = 0.05 * = 0.1 NS/(empty) = > 0.1

Covariates

Gender dummy, marital status dummy, two educational level dummies, and five age dummies (Ref.: 36–45 years)

Only the coefficients for 2011 and the interaction term LLSI × 2011 is shown. Full models available on request

## Abbreviations

EU-SILC: European Union Statistics on Income and Living Conditions
ILO: International Labor Organization
LLSI: limiting, longstanding illness
OLS: ordinary least squares regression
SRH: self-rated general health
